# Practical Medicine

**Published:** 1878-09

**Authors:** 


### Practical Medicine.

Etiology of Tuberculosis.—Discussion at Fiftieth Congress of German Naturalists in Munich. Session of 21st September, 1877. (*Allgem. med. Centralzeitung*, Nos. 38–45, 1878.)

Prof. v. Buhl made some introductory remarks on the lymph-vessels of the pulmonary parenchyma. Siborsky had previously injected a solution of carmine into the lungs of a living rabbit, and found after death an injected network with stellate openings in the alveolar wall. A repetition of that experiment by Buhl's assistant, Dr. Schweninger, did not succeed. But a better mode of demonstrating the openings of the lymph-vessels consisted in filling a human lung (after death) with a colored fluid. On injecting a fluid containing cinnabar in suspension into the lungs of a living dog, and killing the dog at once by ligating the trachea, the cinnabar passes completely into the lymph-vessels and none remains in the alveoli.

Microscopical examination of such lungs shows conclusively that the lymph-vessels open into the alveoli. These openings are evidently dilated during inspiration, facilitating the entrance of any alveolar contents, solid or fluid, while the compression of these apertures during expiration prevents the expulsion of any particles.

In Dr. Buhl's institute, Dr. Lippl experimented on the direct inoculability of phthisis. As subjects he chose dogs, on account of their relative immunity from that disease. Drying and pulverizing some tuberculous sputa, he caused this dust to be inhaled by a dog through an opening in the trachea. This was repeated on three days; the animal died the twelfth day. The autopsy revealed numerous white miliary nodules in the lungs, on the pleura and peritoneum, but they were not tuberculous, consisting simply of fat molecules and fat drops. But a decided miliary tuberculosis of the lungs was obtained by introducing into the lungs 30 to 50 C. C. of an emulsion of sputa with a 1 per cent. solution of salt. On repeating the inhalation two to four times and keeping the dogs alive for about two months, a success was obtained in every case.

Further experiments were reported by Dr. Tappeiner. He placed three healthy dogs into large, airy kennels. Taking a spoonful of fresh tuberculous sputa, he made an emulsion of it with 900 gr. of a 1 per cent. solution of Na Cl. This fluid was put into a steam atomizer in the kennels for one to two hours daily. The dogs remained apparently healthy, but on killing them after four weeks, a true disseminated miliary tuberculosis was found. Two other dogs receiving daily two spoonsful of the same sputa in their food, presented likewise a genuine miliary tuberculosis after six weeks, without having shown any symptoms during life. The innoculability through pulmonary and intestinal absorption was thus positively proven in animals noted for their immunity from spontaneous phthisis.

Dr. Schweninger, who had made the microscopical examination in the above cases, hereupon demonstrated the specimens, proving satisfactorily their tuberculous nature.

Speaking about the identity of human and animal tuberculosis, Prof. Bollinger criticised it as a mistake to employ rabbits and guinea-pigs for inoculation, since these amimals easily acquire tubercular affections from the most various (non-specific) causes.

On the other hand, having injected caseous masses from a tuberculous tongue of a cow into the peritoneal cavity of a goat, he found that animal presenting a genuine miliary tuberculosis of the peritoneum after the lapse of six weeks. Prof. B. further discussed the relation of phthisis to scrofula. By inoculating a goat in the peritoneum with some scrofulous gland-matter removed from the axilla of a man, an exquisite miliary tuberculosis of the peritoneum was obtained. Subsequent examination revealed an initial pulmonary infiltration in that human patient. On the other hand, the feeding with true tuberculous matter will produce in some animals a scrofulous bowel affection, bearing a complete resemblance to the tabes meseraica of cattle.

Prof. Ponfick attempted to follow the mode of propagation of the tubercular virus, by examining the thoracic duct in all autopsies. In all cases of a strictly localized tuberculosis, the duct was found intact. But in the majority of instances of disseminated tubercles there was found a multiple eruption of miliary or aggregated tubercular nodules in the internal coat of the thoracic duct. This appearance is a manifestation of the passage of an impure irritating lymph ; the impurities, however, we know only by their effects.

Omitting the purely critical part of Prof. Kleb's remarks, we will mention only his new results and views. In order to determine whether the contagious agent was the cells, of which the tubercle consists, or some other multiplying virus, he made some experiments in cultivating and isolating the virus. Placing some tuberculous matter in pure albumen of the egg, protected from dust, he found the latter soon cloudy from the presence of numerous exceedingly small molecules and short rods. On placing a drop of this mixture into successive portions of fresh albumen, the original substance of the tubercle was soon virtually removed by the increasing dilution ; the molecules, however, continued to multiply.

*Albumen thus inoculated, can produce true tubercles when introduced into the body of a healthy animal.* On the other hand these fine molecules, if really the bearer of the virus, ought to be found in all tubercles, and Klebs did actually succeed in finding them. In all tubercles he examined he observed an immense number of molecules of barely visible dimensions, measuring 0.2, 0.5 micromillimeters. They were in constant movement, which movement was proven to be of vital origin by its cessation in high temperatures. The molecules were seen most abundantly in cases of acute disease in parts in which discrete tubercles were not yet found.

In the mesentery of a cat inoculated with albumen containing tubercular virus, the tubercles were found distributed along the course of the vessels. They consisted of proliferated endothelial cells, and contained but few white corpuscles. Emigration of leucocytes is only an accidental occurrence in the development of tubercle. Between the endothelial cells the molecules previously described were found. But in and around the walls of vessels—at spots where no tubercles existed as yet, these same molecules were found likewise in accumulations. Prof. Klebs does not hesitate to call these corpuscles the bearers of the tuberclar virus, and to consider such accumulations along the vessels as the starting point of tubercles.

COLLODION IN SEA-SICKESS. Dr. Laederich. (*L'Année Médicale*, June, 1878, p. 101.)

The author has seen many cases in which sea-sickness has been prevented by the use of collodion as described below. He gives two striking cases in which the patients, who had previously suffered from sea-sickness every time they had crossed the ocean, were entirely prevented from it by the collodion.

The author's mode of using the collodion is as follows:

Before embarking, the traveler applies successively three coats of ricinated collodion with a brush over the epigastric region, not extending beyond the anatomical limits, which he indicates thus to persons consulting him: a horizontal line just above the umbilicus, to the right and left two vertical lines situated 5 centimeters outside of verticals, let fall from the nipples; finally, above, a line parallel with the costal borders, and 3 or 4 centimeters above these. When the journey is to be of several days' duration, he recommends the traveler to take a supply of collodion with him to repair the cracks sometimes produced in the coating.

The author has no proofs of this method being curative of cases already begun, but believes it would be so. He does not pretend to explain the action of collodion in these cases. He only knows that it is a powerful anti-emetic when thus applied, and has employed it in other cases, such as traumatic peritonitis, in which frequent and painful vomiting has been either entirely arrested or very much relieved, and the patients very much comforted. For this reason, he thinks it might be curative of sea-sickness as well as preventive.

Bryonia and Drasera in the Treatment of Whooping-Cough.—Dr. Louvet-Lamare. (*L'Année Médicale*, June, 1878, p. 105.)

The author gives in the first or catarrhal stage of the disease a daily dose of 1 gram of the tincture of bryonia to a child of seven years. The medicine does not abridge this stage, but it very sensibly diminishes the tracheo-bronchitis, which is then the only morbid manifestation ; moreover, being bitter, it stimulates the appetite, and does not occasion nausea.

When the characteristic cough occurs, he gives the tincture of drasera in daily doses of 1 gram for a child of seven years, and the frequence and violence of the coughing fits at once diminish. The value of this treatment has been proved in a large number of cases, and also in the fits of coughing, in certain cases of bronchitis, and even in phthisis, though not so marked as in pertussis. But little success has been obtained from it in coughs caused by laryngitis and laryngo-trachitis.

The dose may be increased by 5 drops or more every second day without danger, as it is not poisonous. The cough diminishes still more, but usually lasts for two or three weeks before convalescence. If, on the contrary it be given to a child who has had the cough for three weeks or so, one may expect to *cure the worst cases in a few days.* From a large number of cases thus treated, the author concludes that the disease may be shortened to at least one half the time required by change of air, which is often impossible.

Tapping a Vomica in Phthisis.—Williams.—(*Br. Med. Jour.*, July 20, 1878.)

A patient aged twenty-eight, had had consumptive disease for six years. A large cavity with pleuritic adhesions had formed at the base of the left lung, besides others in other parts. The cough was harrassing, the expectoration very profuse (nearly a litre every 24 hours), and very offensive. Microscopical examination showed that lung degeneration was rapidly advancing. At the writer's request, Mr. Erichsen introduced a large-sized trocar and cannula between the seventh and eighth ribs, and withdrew a litre of fœtid pus ; the operation being somewhat difficult on account of the toughness of the fibrotic lung. Relief was immediate, all fœtor ceased, both pulse and temperature fell, and the expectoration was reduced to 60-100 C.C. Limited pneumothorax and cutaneous emphysema followed, the latter soon subsiding. Even at the expiration of a month, the discharge was slight. The cavity had several times been washed out with antiseptic fluids.

The operation had previously been done in this country by Dr. Pepper, in England by Dr. Ramadge, in Germany by Dr. Mosler, and by others. The practice is only suited to exceptional cases, such as those of fœtid expectoration. It has been recommended to accomplish the same purpose by catheterisation of the air-passages, by large thoracentesis, by aspiration, by caustic and by galvano-caustic, the latter preferred by Koch of Berlin.

